# Exploring the High-Grade and Refractory Neurotoxicity of Teclistamab: An Underreported Entity

**DOI:** 10.7759/cureus.49192

**Published:** 2023-11-21

**Authors:** Forat Lutfi, Al-Ola Abdallah, Muhammad Nashatizadeh, Nausheen Ahmed, Maggie Nelson, Janna Hamideh, Zahra Mahmoudjafari, Leyla Shune

**Affiliations:** 1 Hematologic Malignancies and Cellular Therapeutics, University of Kansas Medical Center, Kansas City, USA; 2 Neurology, University of Kansas Medical Center, Kansas City, USA; 3 Oncology, University of Florida, Gainesville, USA

**Keywords:** immune repose, chemotherapy toxicities, newest treatment for multiple myeloma, bispecific t cell engager, immune effector cell-associated neurotoxicity syndrome (icans)

## Abstract

T-cell re-directing bispecific antibodies targeting B-cell maturation antigens have recently entered real-world use in relapsed/refractory multiple myeloma. While no head-to-head comparison has been done, they have generally been observed to have lower-grade toxicities compared with their chimeric antigen receptor T-cell (CAR-T) counterparts. However, in our real-world, single-institution experience, we have encountered two patients receiving teclistamab who experienced high-grade and refractory immune effector cell-associated neurotoxicity syndrome (ICANS) that did not respond to traditional toxicity mitigation strategies of high-dose corticosteroids or other immunosuppressive therapies. As we increase our use of these novel and vital agents, caution must be warranted.

## Introduction

The multiple myeloma (MM) therapeutic armamentarium has continued to expand rapidly in relapsed/refractory multiple myeloma (RRMM), with particular excitement in the T-cell re-directing bispecific antibody targeting B-cell maturation antigen (BCMA) [1]. The phase I/II MajesTEC-1 study of RRMM patients treated with teclistamab demonstrated a response rate of 63% and a complete response rate of 39.4%, with median progression-free survival and overall survival of 11.3 and 18.3 months, respectively. High-grade (≥ grade three) cytokine release syndrome (CRS) or immune effector cell-associated neurotoxicity syndrome (ICANS) were reported in 0.6% and 0, respectively. These findings led to FDA approval for treatment in RRMM [2,3].

We report two unusual cases of RRMM treated with teclistamab with persistent and high-grade neurotoxicity. Our two patients were classified as refractory ICANS since they did not respond to traditional toxicity mitigation strategies employed for bispecific antibodies and chimeric antigen receptor T-cell (CAR-T) therapies. Herein, we provide insight into both cases, the development of ICANS, treatment strategies employed, and finally, discuss the future directions that may be considered. To our knowledge, this is the first report of refractory ICANS secondary to teclistamab in RRMM to date.

## Case presentation

Both patients had high-risk cytogenetics, received at least six lines of previous therapy, and were at least triple class (anti-CD38, proteasome inhibitor, and immunomodulatory drugs) RRMM, with a duration of less than 24 months since diagnosis. These disease characteristics are suggestive of more virulent disease biology when compared with MajesTEC-1 trial patients (see Table 1). Both patients had multiple comorbidities. However, neither had a history of baseline neurologic disease or CNS MM involvement, aside from a remote history of TIA in patient B. Both patients had a baseline Immune Effector Cell-Associated Encephalopathy (ICE) score of 10 out of 10.

**Table 1:** Clinical Characteristics and Outcomes *All values given in MajesTEC-1 study are the median, unless otherwise noted. AutoHSCT= autologous hematopoietic stem cell transplant, RRMM = relapsed refractory multiple myeloma, CRS = cytokine release syndrome, ICANS = immune effector cell associated neurotoxicity syndrome CRS grading was done using Lee criteria and ICANS grading was done using the American Society for Transplantation and Cellular Therapy (ASTCT) grading criteria.

	MajesTEC-1* (n=165)	Patient A	Patient B
Age, in years	64	64	66
Time since diagnosis, in years	6.0	2	0.7
High-risk cytogenetics	26%	Yes	Yes
Extramedullary plasmacytoma	20%	Yes (pleural effusion, muscle)	No
≥60% plasma cells in bone marrow	11%	Yes	Yes
Previous lines of therapy	5	7	6
Previous autoHSCT	82%	No	No
Triple class RRMM	78%	Yes	Yes
Refractory to last line of therapy	90%	Yes	Yes
CRS Grade III/IV	0.6%	No	Yes
ICANS Grade III/IV	0	Yes	No

**Figure 1:**
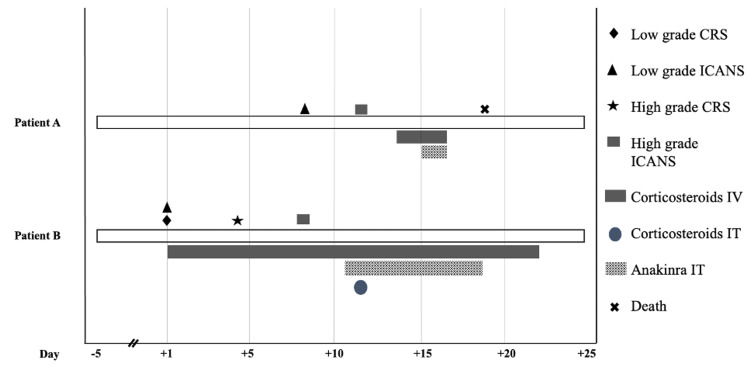
ICANS Timeline and Interventions CRS = cytokine release syndrome, ICANS = immune effector cell associated neurotoxicity syndrome, IV = intravenous, IT = intrathecal

Patient A completed cycle one (0.06-0.3-1.5mg/kg infusions) of teclistamab developing ICANS grade one with an ICE score of 7 out of 10 on day 8 which was initially attributed to overall fatigue and procedures (see Figure 1). ICE scores improved spontaneously to 9 out of 10. However, on day 12 he declined further to ICANS grade III with ICE scores of 1 out of 10. Inflammatory markers were elevated with peak concentrations at day 12 with ferritin of 5,486ng/mL (pre-treatment baseline was 1715ng/mL) and c-reactive protein (CRP) of 3.21mg/dL (pre-treatment baseline was <1mg/dL). He was started on dexamethasone 10mg intravenously (IV) every six hours on day 14 and IL-1 antagonist anakinra 100mg IV twice daily on day 15 for severe ICANS. A computed tomography (CT) scan of the head on day 16 (delayed due to instability) did not reveal any mass effect or hemorrhage. There was concern for possible metabolic encephalopathy given uremia, but the patient did not improve following multiple sessions of dialysis. Unfortunately, he had no response to dexamethasone or anakinra and passed on day 18 from refractory ICANS and multiple organ failure.

Patient B received the first step-up dose (0.06mg/kg) of cycle one and within hours experienced hypotension and tachycardia and cognitive impairment consistent with CRS grade I and ICANS grade I and was started on dexamethasone 10mg IV daily. She continued to decompensate and experienced circulatory shock requiring vasopressors on day 4 consistent with CRS grade III and ongoing ICANS grade I. The circulatory shock resolved within 24 hours on a higher dose of dexamethasone 20mg IV every six hours. However, her ICANS worsened to grade III on day 8. Full neurologic workup was non-revealing with electroencephalogram (EEG) findings of abundant sharply contoured waves of triphasic morphology and generalized delta greater than theta slowing, consistent with moderate non-specific encephalopathy without evidence of epileptiform activity, three magnetic resonance imagings (MRIs) of the brain without any parenchymal signal abnormality or enhancement to suggest neurotoxicity, and two lumbar punctures without any notable findings. Serum ammonia was mildly elevated in the absence of liver disease which led to the initiation of rifaximin without improvement. Given this lack of response and severe ICANS, we administered hydrocortisone 25mg intrathecally on day 12, anakinra 100mg IV twice daily from days 11 to 18, and methylprednisolone 1000mg IV from days 15 to 21. Inflammatory markers were notably elevated with peak ferritin of 1621ng/mL (pre-treatment baseline 709ng/mL) on day 16 and CRP remaining normal throughout. Interestingly, she achieved a brief partial response after a single step-up teclistamab injection. At the last follow-up on day 29, the patient remained with ICANS grade III and passed away due to progressive disease on day 31.

## Discussion

Although no head-to-head comparison has been done, T-cell re-directing bispecific antibodies have typically been observed to have lower grade CRS and ICANS compared with their CAR-T counterparts, making them quite appealing, particularly in outpatient community practices. However, in our recent real-world, single-institution experience of 28 patients with RRMM treated with teclistamab, we encountered two patients (7%) with refractory ICANS which was not reported in the MajesTEC-1 trial. As teclistamab begins to enter greater clinical use, we believe it imperative to report these early findings to initiate a broad discussion and prepare other clinicians and centers for this possibility, however, rare it may be. Certainly, these two patients had notable comorbidities and aggressive disease biology increasing the risk of toxicity, however, importantly, they would not have been excluded based on trial inclusion and exclusion criteria and are reflective of a subset of patients treated with teclistamab in the real-world [4,5]. There are likely specific factors in these two patients that rendered such severe toxicities after just one cycle in patient A and a single 0.06 mg/kg step-up dose in patient B. It remains to be seen if this includes baseline organ dysfunction (unknown pharmacokinetics in severe renal or hepatic impairment), intrinsic BCMA-directed T-cell re-directing bispecific antibody behavior, native T-cell and other innate/adaptive immunophenotypes, baseline and evolving cytokine milieu, myeloma disease characteristics (disease burden, extramedullary disease), and variations in BCMA expression and in particular on target, off myeloma binding in the CNS [6,7]. CNS expression of BCMA in neural and support tissue as a source of on-target, off-myeloma neurotoxicity remains debated with conflicting findings. In mouse models, BCMA targeting antibodies had a deleterious impact on axonal elongation, while in a study of healthy adults, BCMA was been found to be negligibly expressed by both immunohistochemistry and RNA-sequencing [8,9]. However, in a patient developing Parkinsonism in the CARTITUDE-1 trial, BCMA expression was noted on neurons and astrocytes in the patient’s basal ganglia [10]. This perhaps suggests heterogeneity in CNS BCMA expression amongst patient populations, which may reflect the patchy incidence of severe ICANS in BCMA-targeted therapies. Importantly, in our two patients, neurologic imaging did not have any abnormalities despite severe ICANS. This contrasts with severe ICANS reported in the literature and our own experience following CD19 and BCMA-directed CAR-T therapy where T2/FLAIR parenchymal hyperintensity, cerebral edema, or midline shift may be seen [11,12].

## Conclusions

Unlike the CRS and ICANS, we have seen with CAR-T therapy and even that reported in MajesTEC-1, these two patients did not respond to high-dose steroids, anakinra, or intrathecal steroids as we would expect. This suggests the possibility of a different mechanism of action for these toxicities, although certainly still in the context of a hyper-inflammatory environment with both patients having grossly elevated ferritin. Thus, in the future, novel approaches to toxicity management must be considered and in particular further clinical and translational research is needed to better understand the underlying etiology and pathogenesis of these toxicities. As we increase our use of teclistamab and other bispecifics while caution must be warranted, they remain vital in the RRMM setting, and in time we will learn their appropriate use and management of their toxicities as they arise. Much remains to be learned in the management of these rare but profound toxicities.
